# Supplementary material for: Tailored Triggering of High-Quality Multi-Dimensional Coupled Topological States in Valley Photonic Crystals
Source: Nanomaterials (Basel). 2024 May 19;14(10):885. doi: 10.3390/nano14100885 (PMC11123996; doi:10.3390/nano14100885)
Supplement: Supplementary file 1 [file nanomaterials-14-00885-s001.zip › nanomaterials-2940224-supplementary.pdf]

Supplementary Materials for Tailored Triggering of High-quality Multi-dimensional Coupled Topological States in Valley Photonic Crystals

Guangxu Su[1†], Jiangle He[1†], Xiaofei Ye[2], Hengming Yao[1], Yaxuan Li[1], Junzheng Hu[2], Minghui Lu[3], Peng Zhan[2*] and Fanxin Liu[1*]*

[1]Department of Applied Physics, Zhejiang University of Technology, Hangzhou 310023, China

[2]National Laboratory of Solid State Microstructures, Collaborative Innovation Center of Advanced Microstructures, School of Physics, Nanjing University, Nanjing 210093, China

[3]National Laboratory of Solid State Microstructures, Department of Materials Science and Engineering, Nanjing University, Jiangsu Key Laboratory of Artificial Functional Materials, Nanjing 210093, China

* Corresponding author: liufanxin@zjut.edu.cn, and luminghui@nju.edu.cn, and zhanpeng@nju.edu.cn.

Fig. S1. Valley-momentum locking phenomenon of two edge states at the K' valley.

Fig. S2. Field distribution of different hybrid modes in transmission spectrum.

Fig. S3. Excitation of high-quality tunable multi-dimensional coupled topological states with LCP source.

Fig. S4. Robustness of multi-dimensional coupled topological states.

Fig. S5. Regulation of multi-dimensional coupled topological states with different coupling strength when the RCP source is at position 3.

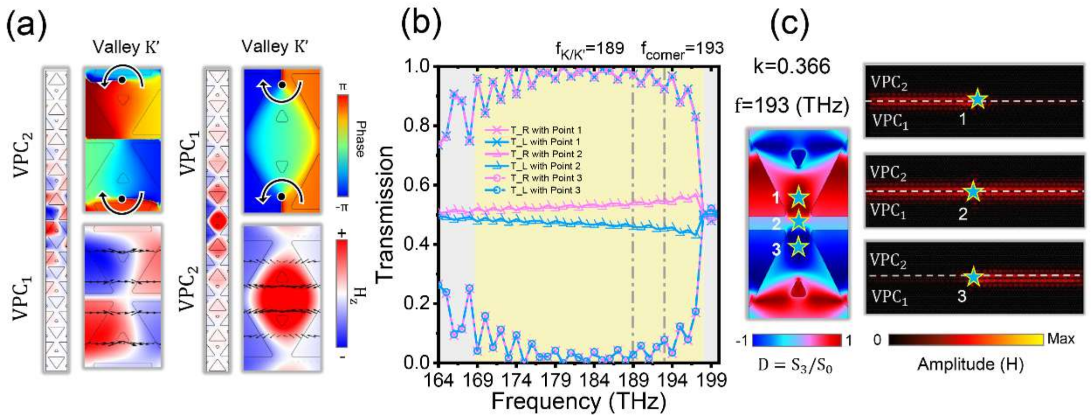

Fig. S1. Valley-momentum locking phenomenon of two edge states at the K' valley. (**a**) Valley-momentum locking properties of type I and type II VESs at K'-valley. (**b**) Normalized energy flow at the left or right ports when a LCP source is at positions 1, 2, and 3, respectively. (**c**) Positional dependence of the normalized Stokes S_3/S_0 parameter at the interface, and the field distributions with the LCP source at positions 1, 2, and 3.

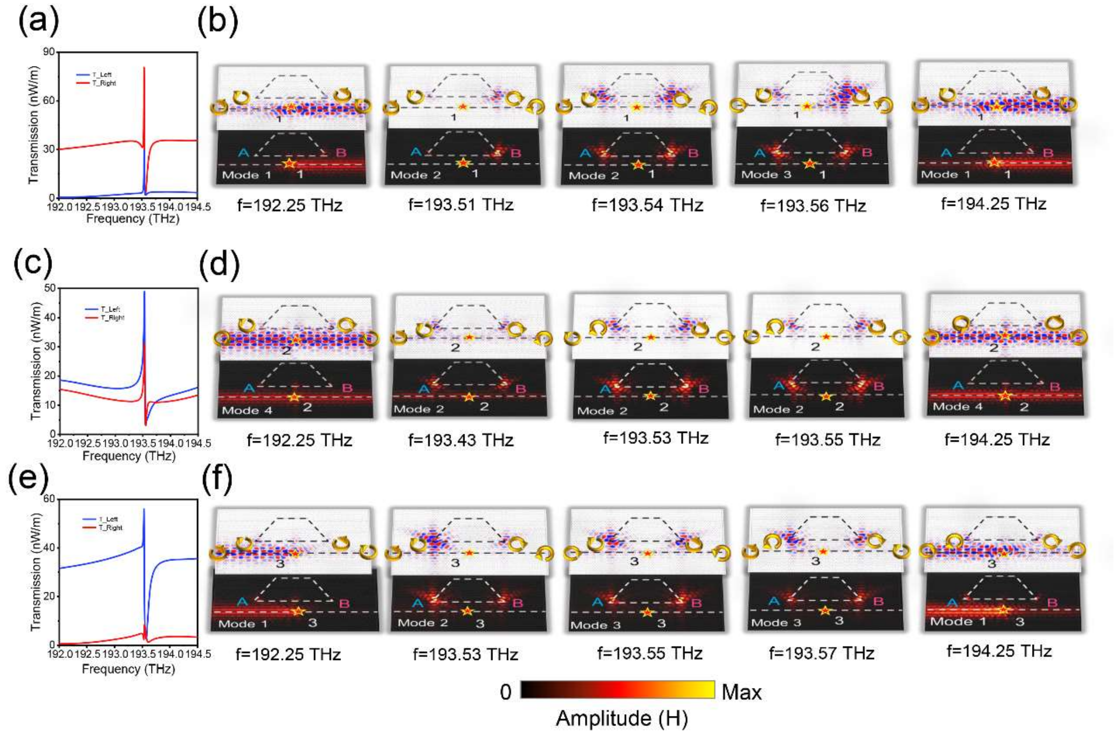

Fig. S2. Field distribution of different hybrid modes in transmission spectrum. (**a-f**) H and H_z distributions of modes at different frequencies in the transmission spectral line when the RCP source is at positions 1, 2, and 3, respectively, where the dark modes are $Mode_2$ or $Mode_3$ and the bright modes are $Mode_1$ or $Mode_4$.

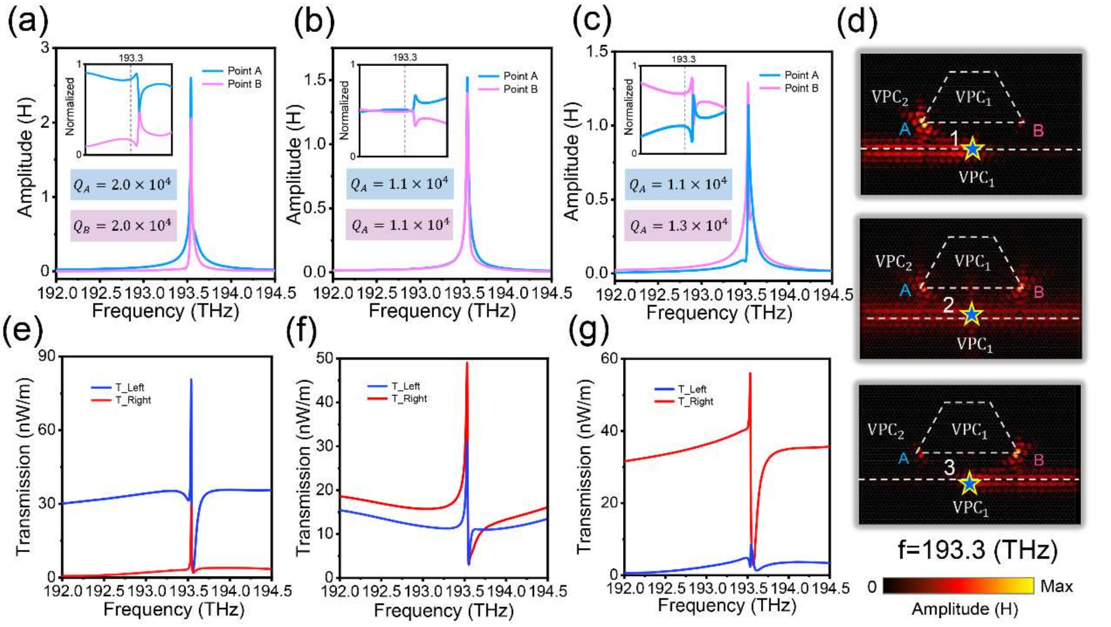

Fig. S3. Excitation of high-quality tunable multi-dimensional coupled topological states with LCP source. (**a**)-(**c**) Field intensity of the VCS nanocavities when the LCP source is at positions 1, 2, and 3, respectively, and the inset shows the normalized intensity at points A and B. (**d**) Field distributions of the LCP source at the 1, 2 and 3 positions, respectively. (**e**)-(**f**) Transmission spectra of the two ports of the waveguide when the LCP source is at positions 1, 2 and 3, respectively.

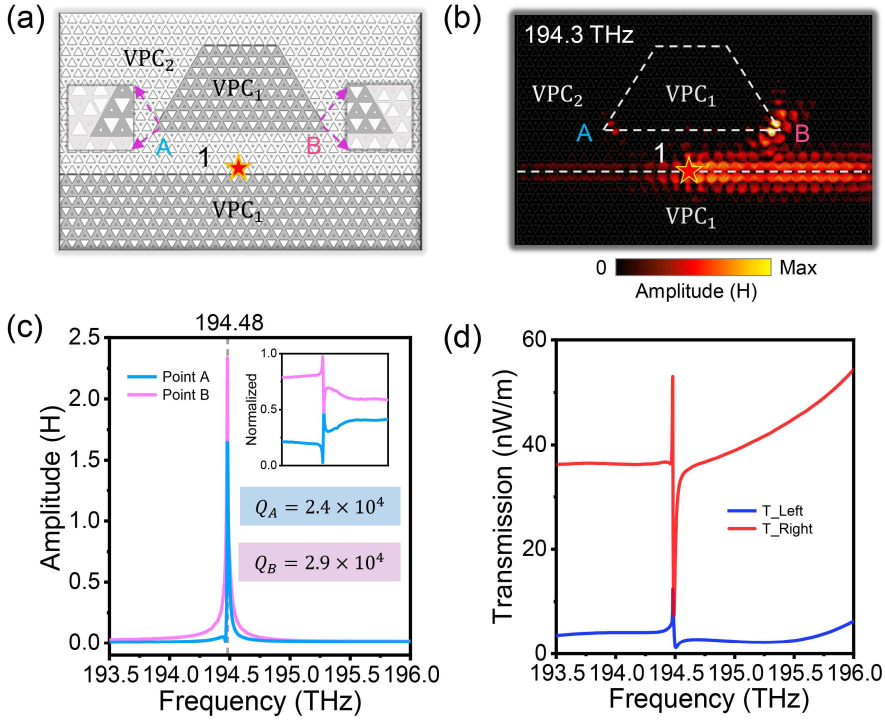

Fig. S4. Robustness of multi-dimensional coupled topological states. (**a**) Schematic of the structure, where a small triangular hole near the splicing corner is replaced by a large triangular hole as shown in the enlarged figure. (**b**) Field distribution of the multi-dimensional coupled topological states with defect. (**c**) Field intensity at points A or B, and the inset shows the normalized intensity. (**d**) Transmission spectrum at the left or right ports.

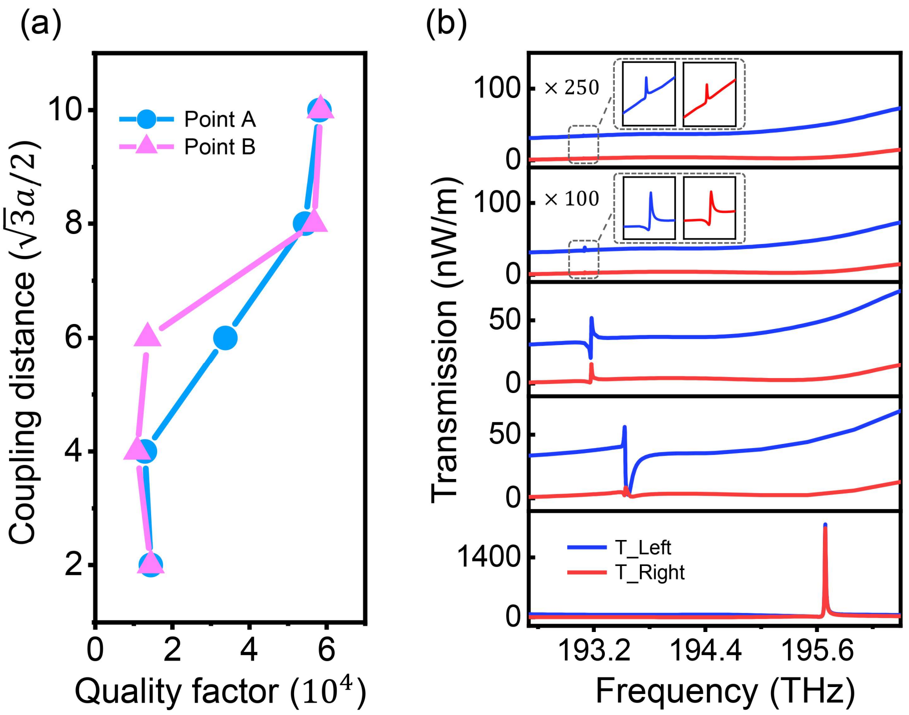

Fig. S5. Regulation of multi-dimensional coupled topological states with different coupling strength when the RCP source is at position 3. (**a**) and (**b**) Dependence of the quality factor and transmission spectrum of the structure with different coupling strength when the RCP source is at positions 3.
